# Using Recontextualisation Theory to Understand Learning Across Multiple Sites in Simulation‐Based Nurse Education

**DOI:** 10.1111/jan.70163

**Published:** 2025-09-01

**Authors:** Helen T. Allan, Mike O'Driscoll

**Affiliations:** ^1^ Faculty of Health, Social Care and Education Middlesex University London UK

**Keywords:** clinical placements, knowledge recontextualisation, nurse education, simulated learning, simulation

## Abstract

**Aim:**

The aim of this discussion paper is to explore whether recontextualisation theory deepens our understanding of learning across multiple sites when introducing simulation‐based education (SBE) into nurse education.

**Background:**

The requirement for students to learn in clinical placements remains an aspiration as well as a regulatory requirement internationally. Yet, the increasing complexity of healthcare and the numbers of vacancies in the healthcare workforce globally have led to poor learning environments. In the context of faster internet speeds, rapid development in virtual technologies, affordability of hardware, and the move to online educational provision after the COVID‐19 pandemic, SBE has emerged as a key teaching method in health professional preparation programmes globally.

**Design:**

Critical discussion paper.

**Methods:**

This discussion paper is based on current literature on SBE and recontextualisation theory.

**Findings:**

Evaluations of SBE often show positive outcomes for learning in nurse education. Weaknesses and gaps in the evidence on SBE, such as the scarcity of control groups or longitudinal studies, have been identified. Using recontextualization theory, we argue that SBE may also increase the theory‐practice split for students across multiple sites of learning.

**Conclusions:**

The introduction of SBE offers supplementary positive learning opportunities to those in clinical practice while at the same time creating multiple sites of learning which are not always aligned. More needs to be done to teach from a curriculum which relies on students being motivated and able to learn across multiple sites of learning.

**Implications for the Profession and Patient Care:**

To support student nurses in UG professional preparation programmes which rely on SBE as well as clinical practice and universities, shared values between nurse educators and clinical nurses need to be enacted collaboratively. This could be achieved by reframing how students and nurses learn and rework knowledge across sites of learning.

## 1 Introduction

In this paper we discuss whether recontextualisation theory deepens our understanding of learning across multiple sites when introducing simulation based education (SBE) into nurse education. It continues work by Rooney and Nystrom (2018, 53) which ‘problematises and disrupts homogeneous understandings of the simulation space as found in much of the health sciences literature’. Recent stresses on health systems have led to a rethinking about how and where healthcare students learn by both universities and health organisations (Cook et al. 2011; Waxman et al. 2019). SBE has been seen as a partial replacement for clinical placements (Rochester et al. 2012) which serves as a bridge between theory and work (Gonzi 2013; Hopwood et al. 2016).

## 2 Background

Research into pedagogies in nurse education, and clinical learning and supervision in practice environments globally is growing (O'Connor 2024). Nurse teachers draw on [among others (Pagnucci et al. 2015)]:

- Vygotsky's ideas around situated learning and the relationship between learner and supervisor or mentor and peers (Abdal‐Haqq 1999).
- Dewey's ideas on reflection as ‘an active process that involves forming hypotheses and trying them out here and now in the real world’ (Dewey 1916, 115 cited by Rolfe (2014, 1179/80)).
- Eraut's work on tacit knowing (Eraut 2000) validated and integrated practice knowledge (Rutter 2009) in professional education curricula. Eraut's work gave more focus to students' learning across different clinical areas and between academic sites of learning and practice. Informed by these theories, nurse teachers adopted learning approaches in curricula to produce active learners who develop into critical and reflective graduates delivering person‐centred care (O'Connor 2024).

These theories have in common a view of learning as a social and emotional process that is enhanced within the real (clinical practice) environment (Gonzi 2013). These same approaches underpin SBE (Jackson et al. 2024). Curricula (across university and clinical placements) provide learning opportunities that allow students to develop active learning and integrate both theoretical and practical forms of knowledge.

### 2.1 Data Sources

We draw on published research and our research experience in the field of nurse education and SBE to discuss current evidence on the role of SBE in nurse education. Data sources included peer‐reviewed and research literature from a search of PubMed, MEDLINE, Scopus, Embase, ScienceDirect, Web of Science and CINAHL databases to generate literature from 2010 to 2025 that captured research and/or debates before and after the expansion of SBE in nurse education. Key words were: SBE, nurs* education, evaluation, learning. 20 peer‐reviewed research articles were retrieved in two searches, and further sources were located through a search of the grey literature. The majority of the papers reviewed were either literature reviews or theoretical, i.e., no primary data; the remaining papers used mainly quantitative methods. The authors were international from Australia, New Zealand, Europe, UK and USA.

Evidence supports the use of SBE in addition to technology‐enhanced simulation as a way to supplement learning for procedural training (Morgan 2006), to develop clinical reasoning (Cant and Cooper 2014), decision‐making skills (Stayt 2012; Jackson et al. 2024), critical thinking (Chernikova et al. 2020) and situation awareness (Priambodo et al. 2022). In relation to knowledge and skills acquisition, in a systematic review, Alharbi et al. (2024) found that SBE supports skills and knowledge acquisition. They show that while short‐term retention of skills and knowledge held up, the question of long‐term retention needs further investigation. Evidence for aspects of SBE is lacking and there is insufficient research about emotions in the literature on SBE in nurse education. Despite this, SBE offers flexibility, affordability and accessibility to educational providers (Bauman 2016) and has been used in undergraduate nursing education for nearly 30 years, complementing traditional teaching methods (Foronda et al. 2013; Jackson et al. 2024). There are also a number of weaknesses and gaps in the evidence on SBE, such as the scarcity of control groups or longitudinal studies (which focus on the retention of acquired skills gained through SBE) (Alharbi et al. 2024).

Learning in SBE is predicated upon role‐play, repeated psychomotor practice, observation and feedback (Haskvitz and Koop 2004) as well as cognitive apprenticeship, embodied learning, practice theory, gradual release of responsibility, problem‐solving, authentic task assessment and scenario‐based learning (Ledger and Fischetti 2019). Simulation is presumed to provide mock experiences (Wellard et al. 2007) which may have low to high fidelity (closer to real‐life experience). Fidelity has been the *Holy Grail* of SBE in nursing (Rooney and Nystrom 2018) but the evidence for the association of higher fidelity with better learning outcomes is mixed. While the degree of fidelity has been important in the design and use of simulated learning in nursing, it is argued that the affective component of SBE learning has not been given sufficient attention in the literature (Makowski et al. 2017). Yet successful SBE stimulates emotions which then evoke learning (Makowski et al. 2017; DeBlanc and Posner 2022) and these emotional responses to situations and scenarios in SBE need to be recognised and processed in debriefing for learning to be recognised and reflected upon.

Koukourikos et al. (2021) found in their review that SBE provided a protected environment and sense of security which enhances students' self‐esteem and confidence. These experiences aim to represent authenticity using high fidelity in ways that situate the learning experience to and for real‐life clinical practice. While web‐based SBE has shown potential for improved student learning and potential reduction in costs for providers, a significant requirement for SBE is students' self‐regulation in learning (Edelbring and Wahlstron 2016); in other words, their motivation to learn in autonomous learning structures. SBE requires the relationship between student autonomy, flexibility of learning resources, and teacher guidance be acknowledged, planned for and evaluated (Hartley and Bendixen 2001).

Finally, while there is some debate (Waxman et al. 2019), we agree with Erlam et al. (2017) that SBE is not a pedagogy, it is a method of instruction. As such, suitable philosophical paradigms or pedagogical models which assist the teacher to match method (SBE) with desired learning outcomes need to be considered in any teaching (Erlam et al. 2017). Our discussion of recontextualisation theory (Evans et al. 2009) offers a new approach to understanding the role of SBE in nurse education.

### 2.2 Overview of the Issue

There are some significant challenges which are under explored in the literature on SBE, which include the focus of this paper, multiple sites of learning. SBE is effectively another site of learning which student nurses have to navigate and make sense of. Caution has been noted about how much SBE could or should resemble real‐life (Rooney et al. 2015; Hopwood et al. 2016) and whether theory linked explicitly with practice skills in SBE is useful as theory linkages cannot be guaranteed in practice (Bugaj and Nikendei 2016). Indeed, Solomon (2007, 125) argues that SBE is ‘its own kind of real’ which implies that it is not a simple facsimile of practice placements but a distinct learning environment giving experiences that lead to similar or better learning outcomes than placements. This could prove problematic for student nurse learning which is reworked in clinical practice.

Student nurses are required, like medical and other healthcare students, to integrate knowledge across university and different clinical placements even when the knowledge across these multiple sites of learning can seem incompatible to the learner (Ousey and Gallagher 2007; Evans et al. 2010). Rather than a theory‐practice gap (Greenaway et al. 2019), Allan and Evans (2022) conceptualise this gap as a theory‐practice split which arises because teaching in nurse education continues to be predicated upon *applying* theory in or to practice. In addition, despite the approaches described above, students are still sometimes viewed as empty containers who are filled with knowledge in university and transfer this knowledge to practice (Gonzi 2013).

Finally, the requirement for students to learn in clinical placements remains an aspiration as well as a regulatory requirement internationally. Evidence to support either the transfer of knowledge or skills gained through SBE to clinical practice is weak. If SBE were introduced as a partial system response by universities to structural barriers to learning for students in the clinical workplace, the rest of the system for learning i.e., clinical practice has not changed. While the introduction of SBE may have delivered good learning opportunities they are not real life. SBE may be high fidelity and students may show skills and knowledge acquisition in a range of areas, but generally clinical placement capacity in many health care systems has not improved and the increasing complexity of health care, ongoing backlog in patient throughout following COVID‐19 pandemic and the numbers of vacancies in the healthcare workforce globally have led to poor learning environments (Rowland and Trueman 2024). Relationships between clinical practice and nurse education remain complex (Hopwood et al. 2016; Allan and Evans 2022), because clinical nurses, managers and nurse educators have different and contradictory values and aspirations for students.

## 3 Findings

Evaluations of SBE often show positive outcomes for learning in nurse education. Weaknesses and gaps in the evidence on SBE, such as the scarcity of research which utilises control groups or longitudinal studies, have been identified. A more significant weakness in the literature is the lack of evidence that SBE leads to long‐term retention of learning.

Using recontextualization theory, we argue that SBE has constructed yet another theory‐practice split which is unexplored in the literature; SBE is a third learning space, distinct from both university‐based education and learning on clinical placements. This may shape students' learning, and the transfer of learning in skills and knowledge gained in SBE (including both short and long term outcomes) is that SBE constructs another space for learning which requires students to negotiate the social context within those spaces (which may be very different) and the expectations of different actors within those spaces, such as lecturers, assessors, clinical staff, other healthcare professionals and patients (real and mannikins).

In a mixed methods evaluation study, O'Driscoll et al. (2024) showed how student nurses engaged actively in SBE. The pedagogic approach of the SBE evaluated was based on a theoretical framework which was described by the lecturers as constructivism, where learning is constructed by learners rather than knowledge being passively taken in, and where emotions promote learning. Students were encouraged to learn by lecturers throughout the SIMS week design, a mixture of learning online and face‐to‐face through experiences, reflecting on their emotions and through making mistakes. Lecturers led debriefing sessions where students were encouraged to relate personal and professional experiences arising from their learning. Students' engagement was observed in a range of expressed emotions (shock, empathy and laughter) as they reworked knowledge to link theory with practice by reflecting on their learning in SBE in the context of their learning experiences in clinical practice (O'Driscoll et al. 2024). A significant finding was that while SBE is a learning environment, distinct from both university and clinical placements, there was little or no discussion or support for students in navigating between these environments. We now consider what having another site of learning means for nurse education and what this might imply for learning in a professional preparation programme and the theory‐practice split.

### 3.1 Theory‐Practice Split

Allan and Evans (2022) build on earlier work by McKenna and Slevin's (2008) discussion of practice theory and the development of person‐centred nursing theory (McCormack and McCance 2010) to reframe the theory‐practice gap as a theory‐practice split which is continuously reproduced in everyday practice both in academic and clinical nursing. Drawing on Reed (2006) and G. Rolfe (2006), who argue that nurses produce knowledge in practice and Avis & Freshwater's approach to critical reflection, Allan and Evans (2022) do not describe thinking or reflection in practice as *applying* theory in or to practice; instead, learners rework knowledge for practice across different settings Evans et al. (2009). Nurses, like other healthcare professionals, are life‐long, agentic learners who rework knowledge in and through local practice (Allan and Evans 2022). Students (learners) rework or recontextualise knowledge for practice across different settings (Evans et al. 2009)—it is not a linear or one‐way process whereby knowledge acquired in ‘theory’ is ‘applied to practice’. Many students find it difficult to recontextualise knowledge from one sphere of learning to another and there are few resources to support this process. Using recontextualisation theory, Allan and Evans (2022) illustrate how nurses adapt to changes in health care practices as they learn, redefining their roles and building local knowledge to inform practice, thereby reducing the theory‐practice split.

### 3.2 Recontextualising (Reworking) Knowledge as a Framework for Learning

Understanding the flows of knowledge in programmes involving substantial elements of work exposure, such as in nursing and midwifery, goes beyond typologising forms and features of knowledge. Evans et al. (2009) argue that the knowledge logics that underpin programme flows, and how knowledge is changed as it is ‘put to work’ across contexts of learning and practice in universities, colleges and workplaces, need to be understood to produce reworked knowledge that will be useful for practice. All knowledge has a context in which it was originally generated. Contexts are often thought of as settings or places, but contexts in our use extend to the ‘schools of thought’, the traditions and norms of practice, the life experiences in which knowledge of different kinds is generated. For knowledge generated and practised in one context to be put to work in new and different contexts, it has to be recontextualised in various ways that simultaneously engage with and change those practices, traditions and experiences.

Evans et al. (2009) have argued that recontextualisation is a multi‐faceted pedagogic practice, based on the idea that concepts and practice change as we use them in different settings. The original research has drawn on developments of Bernstein's idea that concepts change as they move from their disciplinary origins and become a part of a curriculum (Bernstein 2000; Barnett 2006) and on van Oers' (1998) observation that concepts are integral to practice and change as practice evolves over time and varies from one sector or workplace to another. These notions have been substantially expanded to embrace the ways in which learners themselves change as they recontextualise concepts and practices.

Four sets of practices of recontextualisation are significant in this theory, which are discussed in detail elsewhere: content, pedagogic, workplace, learner (Evans et al. 2009). Recontexualisation theory has been meaningfully applied across several disciplinary fields (Evans 2015) and within nursing (Evans et al. 2010; Allan and Evans 2022). It has not been used to underpin a curriculum in nursing.

## 4 Discussion

Ledger and Fischetti (2019) state that a controlled learning environment such as SBE removes some of the complexity and variance experienced in real‐life clinical settings. While the SIMS week pilot did not seek to replicate placements, and did indeed offer a more controlled and predictable learning environment than real‐life clinical practice, it is still an emotional learning environment which raises complex issues for learners that need to be addressed in debriefing. While it was not ‘real lif’, there was much emphasis on fidelity. This appeared to create an ambiguity between striving for fidelity (to clinical placements) while there are clearly standards that cannot apply in practice such as being allowed to make mistakes. This ambiguity suggests some interesting questions as SBE is another site for nurse education with its own practices and ‘rule’: are students less immersed in the learning because they are aware that it is acceptable to get things wrong in SBE? Is it possible that some students would forget this on placement and think that it is acceptable to make mistakes?

Agatonovic (2023) raises questions about simulation as a learning strategy by asking *what is reality?* He asks us to consider what reality are we teaching our students if SBE is different to clinical practice yet we (effectively) pretend SBE is real, through concerns with fidelity? How do students judge the reality they meet in SBE in comparison with clinical practice? After all, lecturers have a clinical reality from which they construct simulation scenarios but student nurses do not share these clinical experiences, or at least the depth of those experiences. He also questions the purpose of simulation. He argues that SBE ‘implies we can locate ourselves in a simulated centred world that is indistinguishable from a non‐simulated centred world’ (2023, 1583). While SBE replicates certain things that happen or might happen in practice, or things that educators wish would happen or not happen there, students may need the differences between SBE and clinical practice to be more clearly described. Otherwise students might expect the same learning opportunities in practice as they experience in SBE. Finally, we argue that while these students reworked knowledge from clinical practice and university in SBE, our findings did not show whether this learning would be reworked in clinical placements. In constructing another site of learning, SBE, we may have reproduced the theory‐practice split once again in nurse education (Allan and Evans 2022).

Emotions are integral to SBE (Makowski et al. 2017; DeBlanc and Posner 2022); this assumption builds on work in nurse education more widely (Smith 1992; Fabricius 1995). Gonzi argues that:

we need to accept (and take account of this in our teaching and learning approaches) that the complexity of decision making/problem solving in a workplace setting is a holistic process that involves both reasoning and emotions. It is not a cool, rational matter of translating theory into practice. (Gonzi 2002, 1300)

Drawing on Gonzi, we argue that SBE needs to build in, assess and use teaching strategies to develop the reworking of knowledge in students as they transition between learning sites, i.e., academia, clinical placements, SBE, groupwork, assessor relationships (Evans et al. 2009). Our findings show that lecturers and students use opportunities in SBE to recontextualise knowledge from knowledge gained in academia, e.g., the groupwork in SBE based on scenarios, debriefing. But these opportunities in SBE are not evidence that students rework knowledge in practice.

### 4.1 Learning and Nurse Education

It cannot be assumed that learning in the workplace will take place without structure and support or that learners will necessarily be signposted to learning or be able to identify learning opportunities. Learning ecologies need to be actively constructed so that students can establish how to learn in different environments, including those which are not disposed to learning (Jackson and Barnett 2020). Jackson and Barnett (2020, 1) argue that the contemporary world ‘obliges human beings to learn and keep learning across and throughout their lives in order to survive and flourish’. It is debatable whether individual habits of learning can be ingrained and sustained in an organisation such as the NHS. As Evans (2020, 163) argues ‘the ways in which adults learn in the workplace and throughout working life are rooted in occupational contexts and personal biographies’. One of the challenges to our understanding of professional learning is the interaction between the agency of the learning individuals and the structures, institutional processes, milieus and relationships that mediate their continuing learning and development as knowledgeable practitioners. Our findings do not suggest that SBE facilitates this as SBE focuses on the individual learner and the teams they work in rather than the wider organisational context.

As we have described above, the theory‐practice split where learning across different sites appears disintegrated, are well established in nursing. Melia (2006) argued that, while nurse educators often assume that the NHS is a learning organisation, in fact the NHS is primarily focused on patient care. As the lines between public and private are increasingly blurred and neoliberal values have become ingrained in NHS culture (Bayliss 2022) the NHS may often have to focus on financial targets or performance; providing placements or learning support for placements may not be a priority. Perhaps as important, the NHS pays little attention to the informal learning which occurs in the workplace for learners. Evidence suggests that informal learning of poor practice is equally as influential as learning of good practice (Bockarie 2002; Groothuizen et al. 2019). As our findings suggest, as learning is designed to take place over multiple sites, the opportunities for gaps in learning are multiplied and students struggle to rework knowledge between these different sites of learning. The busyness of the NHS, the lack of staff to foster learning and the pressure on work placements where students struggle to rework knowledge through a lack of opportunity (Groothuizen et al. 2019) are other factors which worsen the theory‐practice split. A distinguishing feature of the workplace learning (including the NHS) is its embodied nature (Allan et al. 2018). It is frequently the case that the emotional nature of the work with the lack of time to debrief either about the emotions which may shape that experience or the possible learning from that emotional experience.

Current pedagogical models in many professions fail to focus on knowledge. Quinlan and Song (1998) argue that current models of learning in the workplace are largely a method for the development of competences or motor skills. This has been noted as a risk in SBE (Gonzi 2013). Such models do not focus on the knowledge base required for practice or how a student becomes a professional, a member of a community of practice (Allan and Evans 2022). Neither do they focus on how a student integrates learning across multiple workplaces or sites of learning (Allan and Evans 2022). Practice knowledge, produced and reproduced through practice knowing in local settings for local purposes by individuals and groups of nurses making sense of new roles, challenges the *old* ideas of what nurses should be doing when they nurse. But it also challenges accepted views of what forms of knowledge are valued (Purkis and Bjornsdottir 2006). To enable nurses to produce and value nursing knowledge which emerges in practice, nurse teachers, managers and leaders need to educate nurses to be active learners and co‐producers of knowledge for practice. Practice might then be informed legitimately by theory as nurses contextualise and recontextualise knowledge in new settings with each and every patient. Previous work by Allan and Evans (2022) based on recontextualisation theory focuses on the development of adult vocational learners through exploring the linkages between learning and knowledge production as well as the development of competence and the student's role in the learning process.

In nursing it seems to us that there are fundamental problems with embedding and sustaining an ecology of learning. There are differences between the academy, the curriculum, the national regulator (the NMC), clinical placements and practice workplaces in what they value as learning. They create disjunctions at the meso‐level (O'Toole et al. 2020). These values, meanings and ensuing practices are produced and reproduced through relationships, interactions, objects and methods of learning. Perhaps the most important difference in value is evidenced in the theory‐practice split which is premised on the belief that thinking and doing are different activities; learning is achieved through doing alone. If learning is doing and therefore separated from thinking, wholly embedded in practice itself, then learning in universities and other sites of learning (in SBE, in voluntary work placements, through personal lives, online communities) may not be valued as learning and the learning ecology is therefore under threat because it cannot be sustained in a disintegrated environment.

## 5 Conclusion

We have discussed the significance of SBE as a system response to structural barriers to learning for students in the clinical workplace and the implications of the introduction of SBE as another site of learning in nurse education. We draw on previous writing on the theory‐practice split in nurse education (Allan and Smith 2010; Allan and Evans 2022) to argue that SBE has elements which tend to decrease the theory‐practice split and some which may increase it. The extent to which it can reduce the theory‐practice split depends partly on how SBE is designed and delivered; e.g., giving strong emphasis to debriefing sessions and supporting students to recontextualise knowledge gained in either university or placement settings will tend to decrease the theory‐practice split. While SBE in nursing may increase the theory‐practice split in relationships between university education and professional practices, it may also reduce student anxiety about placements (as shown in our study) and thereby increase confidence about placements, which may help to reduce the theory‐practice split. Further research is needed on the relationship of SBE to the theory‐practice split.

Waxman et al. (2019) states that Chief Nursing Officers feel that new graduates are unprepared to work in their clinical settings, while Deans of Universities feel that departments of nursing prepare their graduates for practice, but that the hospital staff do not adequately support them. Blame is placed on both sides. While academic/service partnerships are critical to the future of nursing education, espoused shared values on the curriculum between nurse education and practice need to be enacted collaboratively. As clinical sites for learning continue to be pressured learning environments, we need to develop good learning opportunities for the next generation of nurses, to remain evidence based about SBE's effectiveness, ensure it is pedagogically not technologically led and with a need for support to recontextualise learning gained in SIMS, in placements and practice.

## Author Contributions

**Helen T. Allan:** conceptualisation, writing – original draft, writing – review and editing. **Mike O'Driscoll:** literature review, writing – review and editing.

## Ethics Statement

The relevant fieldwork permissions were obtained and ethics approval received from Middlesex University, Health & Social Care Ethics Committee in February 2023. Committee application number: 25007. All data utilised in the submitted manuscript have been lawfully acquired in accordance with The Nagoya Protocol on Access to Genetic Resources and the Fair and Equitable Sharing of Benefits Arising from Their Utilisation to the Convention on Biological Diversity.

## Conflicts of Interest

The authors declare no conflicts of interest.

## Data Availability

The data that support the findings of this study are available on request from the corresponding author. The data are not publicly available due to privacy or ethical restrictions.
